# Enhancing E-Nose Performance via Metal-Oxide Based MEMS Sensor Arrays Optimization and Feature Alignment for Drug Classification

**DOI:** 10.3390/s25051480

**Published:** 2025-02-28

**Authors:** Ruiwen Kong, Wenfeng Shen, Yang Gao, Dawu Lv, Ling Ai, Weijie Song, Ruiqin Tan

**Affiliations:** 1 Faculty of Electrical Engineering and Computer Science, Ningbo University, Ningbo 315211, China; kongruiwen@nimte.ac.cn; 2 Center of Materials Science and Optoelectronics Engineering, University of Chinese Academy of Sciences, Beijing 100049, China; lvdawu@nimte.ac.cn (D.L.); ailing@nimte.ac.cn (L.A.); weijiesong@nimte.ac.cn (W.S.); 3 Zhejiang Provincial Engineering Research Center of Energy Optoelectronic Materials and Devices, Ningbo Institute of Material Technology and Engineering, Chinese Academy of Sciences, Ningbo 315201, China; 4 CS-Microsensor (Ningbo) Technology Co., Ltd., Ningbo 311121, China; 5 Research and Manufacturing Base of Olfactory Sensors in China Sensing Valley, Bengbu 233040, China; yanggao@csmsn.com; 6 College of Integrated Circuit Science and Engineering, Hangzhou Dianzi University, Shanghai 201713, China

**Keywords:** electronic nose, MEMS, transfer learning, ReliefF algorithm, feature alignment

## Abstract

This article introduces a novel approach to improve electronic nose classification accuracy by optimizing sensor arrays and aligning features. This involves selecting the best sensor combinations and reducing redundant information for better odor recognition. We employ a feature alignment algorithm to address the discrepancies that impede model sharing between electronic nose devices. Our research focuses on overcoming challenges associated with material selection and the constraints of transferring classification models across different electronic nose devices for drug classification. We fabricated six SnO_2_-based MEMS gas sensors using physical vapor deposition. The ReliefF algorithm was employed to rank and score each sensor’s contribution to drug classification, identifying the optimal sensor array. We then applied feature alignment from transfer learning to enhance model sharing among three inconsistent devices. This study resolves the issue of electronic noses being hard to use on the same database due to hardware inconsistencies in batch production, laying the groundwork for future mass production.

## 1. Introduction

An electronic nose (E-nose) is a gas sensor device designed to mimic the olfactory functions of animal noses. It comprises a gas sensor array, signal preprocessing, and pattern recognition components, enabling both the qualitative and quantitative analysis of gases. The E-nose finds diverse applications across various domains, including food freshness analysis [1,2,3,4], environmental quality monitoring [5,6,7], medical diagnosis [8,9,10,11], explosive detection [12,13], and agricultural inspections [14], among others. One significant application is in the classification of drugs, which is crucial for ensuring quality control, detecting counterfeit products, and enhancing the overall safety of pharmaceutical usage. E-noses offer a rapid, non-destructive, and reliable method for distinguishing between different pharmaceutical compounds, thereby improving efficiency in the pharmaceutical industry.

Advancements in semiconductor technology have led to the manufacturing of electronic components and devices at the micron level. The rise of advanced microelectromechanical systems (MEMS) technology has enabled the integration of micromechanical components [15], electronic components, and sensors. Sensor arrays constructed using MEMS technology possess advantages such as a small size, low power consumption, fast response speed, high accuracy, and strong reliability.

Over the past twenty years, researchers have focused on innovations in E-nose materials [16], signal processing algorithms [17,18], and applications. However, challenges still need to be addressed in the selection of gas sensor materials, as well as issues of reproducibility, which continue to hinder widespread adoption. In classification applications, excess sensors in an E-nose may not be beneficial, and developing targeted sensor materials presents significant challenges. Therefore, selecting high-quality sensors for array optimization is essential.

Another critical challenge is transfer calibration between different E-nose systems. Existing research often focuses on single devices, assuming minimal discrepancies due to device differences. However, in practice, sensor displacement caused by manufacturing processes can lead to signal deviations even among gas-sensitive materials from the same batch, hampering model transferability across devices. Addressing sensor displacement through manufacturing process optimization directly minimizes variability in sensor batches across different devices [19,20]. Although process optimization can control errors within twenty percent, these errors significantly affect results in precision-demanding experiments with low gas concentrations. Consequently, algorithmic calibration on top of process optimization becomes crucial. Zhang et al. successfully solved the drift problem of the electronic nose by combining an Extreme Learning Machine (ELM) with domain adaptation in transfer learning, demonstrating a good calibration performance. However, this success did not extend to the model calibration between different electronic noses [21]. In the model calibration, Wolfrum et al. developed the Partial Least Squares (PLS-2) calibration model, but it was only applied to the concentration calibration of a single gas [22]. Alexander Kononov et al. compared three calibration methods for electronic nose models: UDS (Univariate Data Scaling), DS-L1R (based on L1 regularization), and DS-PLS2 (a multivariate calibration method using partial least squares). The former is more suitable for univariate classification applications, while the latter two are more suitable for multivariate classification applications. In the end, he concluded that UDS is more advantageous for response correction [23].

Inspired by transfer learning, this article simplifies the subspace alignment (SA) method in subspace learning [24]. It proposes a more straightforward and computationally efficient feature alignment (FA) method better suited for calibrating E-nose data. This approach aims to address the hardware consistency requirements in the mass production process of electronic noses, offering a solution to the challenges of variability and reproducibility.

## 2. Materials and Methods

### 2.1. Preparation of the MEMS Gas Sensors

**Figure 1:**
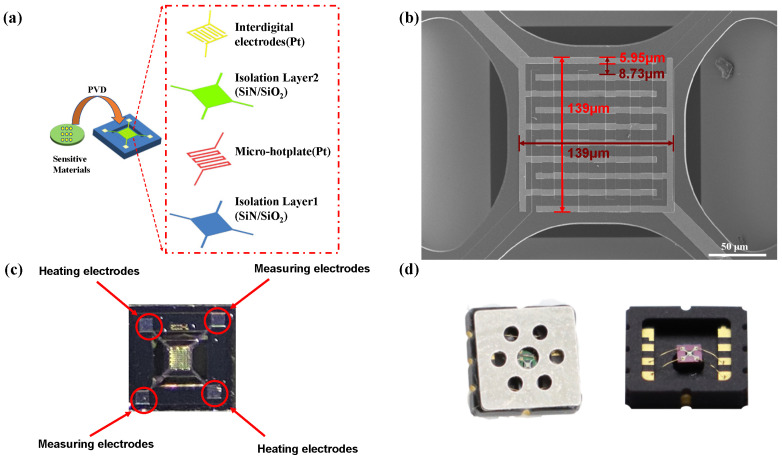
(**a**) Schematic diagram of the structure of MEMS; (**b**) SEM images of the original MEMS substrate; (**c**) Actual image of a MEMS substrate containing two heating electrodes and two measuring electrodes; (**d**) DFN packaging diagram.

The MEMS structure consists of a substrate, an isolation layer, an electrode layer, and a microheater. The bottom isolation layer separates the heating element from the substrate. Meanwhile, the top isolation layer functions as both an insulation layer and a passivation layer between the heater and sensing materials to prevent catalytic interactions between the target gas and the heater material. The microheater provides a heat source and generates temperatures up to 450 °C. Figure 1a schematically shows the structure of the MEMS gas sensor used in this study. The area of the Pt interdigital electrode is 139 × 139 μm in size. The electrode spacing and inter-electrode width were approximately 8 μm and 6 μm, respectively (Figure 1b). At the same time, Figure 1c showed a physical diagram of the MEMS substrate as a whole, which included two heated electrodes to provide a suitable operating temperature for the gas sensor and two measuring electrodes to detect changes in the resistance of the gas-sensitive material.

Physical vapor deposition (PVD) technology was used to prepare pure SnO_2_ sensors and SnO_2_ sensors with different metal species, including Ni, Ag, Cr, Pd, and Pt. Before sputtering, a gold wire can be used to encapsulate the micro hot plate in ceramic packaging, and then the target material can be sputtered onto the micro hot plate. Taking SnO_2_/NiO as an example to illustrate the sputtering process, SnO_2_ and NiO thin films are sequentially deposited on a micro hot plate as oxygen-sensitive thin films. The deposition of NiO or SnO_2_ is performed using an RF sputtering system (HONA Equipment Co., Ltd., China). When depositing the NiO thin film, the deposition duration determines the film thickness. The RF source power is set to 150 W, the gas type is pure argon, and the flow rate is 35.1 sccm. After depositing SnO_2_ for 20 min, a 50 nm thick SnO_2_ film was obtained. The RF source power is 150 W, and the argon flow rate used for NiO deposition during the deposition stage is 37.4 sccm at an ambient temperature. NiO takes 10 min to deposit. The preparation process for other materials is detailed in the Supplementary Materials. Finally, for convenient sensor testing, the prepared sensors were encapsulated using Dual Flat No-leads (DFN) packaging (Figure 1d). After packaging, place the device at a voltage of 1.8 V for heating and aging, which is the process of oxidation

We analyzed the morphology and nanostructure of the SnO_2_ samples using a scanning electron microscope (SEM, S-4800). The crystalline structure of the samples was characterized using an X-ray powder diffractometer (XRD, BRUKER D8 ADVANCE DAVINCl, Germany) with Cu Ka1 radiation (=1.5406).

### 2.2. Construction of Electronic Nose System

#### 2.2.1. Construction of Signal Readout Circuit

The interface circuit is the key to the conversion between the sensor signal and the digital output signal. To a certain extent, the performance of the sensor is determined. At present, the commonly used interface circuits are generally based on analog-to-digital conversion (ADC) technology [25] and pulse-width modulation (PWM) technology [26,27]. In addition to the readout circuit, the heating voltage also has an essential influence on the stability of the sensor. The temperature of the heater fluctuates excessively, which may lead to the drift of the operating point of the gas sensor and consequently affect the detection accuracy. This study used a digital-to-analog converter (ADC)-based technology as the readout circuit, and a low-pass filter was used to reduce signal fluctuations. Next, pulse width modulation (PWM)-based technology was used for pulse heating, and the heating voltage was continuously adjusted through a feedback circuit to make the heating temperature of the sensor more stable.

Figure 1d shows the DFN packaging form. There are eight pins inside the package: one VCC (power), two GND (ground), two heating electrodes (VH), and three measurement electrodes (Vout). The resistance values of sensitive materials in one to three MEMS chips can be loaded and read. Figure S1a shows the packaged chip and its peripheral circuits. Vout 1 and Vout 2 are pins that divide the voltage between the load resistor and the material resistor and are connected to a low-pass filtering circuit to remove noise. The voltage collection point is designed between the load resistor and the material resistor in series to divide the voltage. Then, an ADC chip is used to collect the voltage data of the gas-sensing film and convert the voltage signal into a digital signal to be transmitted to the microcontroller for data processing.

Figure S1b shows a PWM wave control circuit that sets the heating temperature through software. As shown in the figure, the TIM 1-CH 3 pin is connected to the timer output pin of the microcontroller, which can output switch signals of different frequencies and duty cycles (PWM regulation). After passing through a resistor, it is connected to the gate of the MOS transistor. The voltage input end of the MOS transistor is connected to a 3.3 V voltage as the reference for the heating voltage. PWM waves are utilized to control the on–off state of the MOS transistor, thereby regulating the VH terminal to generate various duty cycles and produce distinct heating voltages. This contributes to achieving the goal of setting different heating voltages according to needs. At the same time, a voltage acquisition point VHL 3 is added at this end to connect to the ADC pin of the microcontroller to obtain the current heating voltage synchronously, and noise is filtered out through a second-order filtering circuit to make the voltage value more accurate. This design can accomplish the real-time feedback adjustment of PWM waves, thereby rendering the heating voltage more stable. When the working voltage is 1.5 V, the working temperature of the sensor is around 200 °C.

#### 2.2.2. Construction of Electronic Nose Collection System

**Figure 2:**
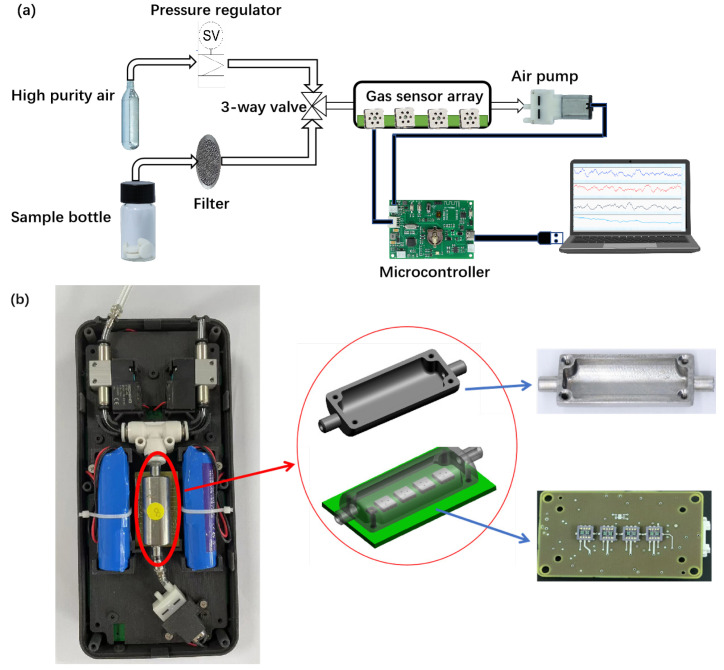
(**a**) The schematic diagram of the electronic nose system; (**b**) The physical image of the electronic nose system.

The schematic diagram of the electronic nose system in this article, shown in Figure 2a, includes a gas source, gas sensors, a microcontroller, and a laptop. The gas sensor array is placed in a small 6 mL chamber to avoid interference from ambient air. The chamber’s internal design includes a curved surface to minimize dead corners, ensuring an optimal gas flow and reducing gas residue. The chamber is made of 304 stainless steel, chosen for its excellent corrosion resistance and durability against corrosive gases. The optical image of the entire electronic nose is depicted in Figure 2b. More views and details can be seen in Figure S2.

The microcontroller is connected to the laptop via a serial port, and real-time data are collected using upper computer software (gas sensor array monitoring and an analysis system based on LabVIEW, as shown in Figure S3). The microcontroller includes switches for the serial port and an air pump. At the front end, high-purity air, test samples, and the chamber are connected through a three-way valve. High-purity air is used to clean the chamber after each test cycle to eliminate external environmental interference.

### 2.3. Testing Methods and Signal Processing

The classification samples used in this study are five drugs purchased from the market, as shown in Table 1. In this experiment, five drugs were classified and stored in 10 mL glass bottles. A physical image of them can be found in Figure S4. The sampling process begins by rinsing the sensor chamber with high-purity air for 10 s. Then, the sample odor is introduced for 5 s, followed by an additional 100 s of rinsing the chamber with high-purity air. For each gas sensor, 15 pieces of raw data will be obtained for each sample, resulting in 75 raw datasets.

**Figure 3:**
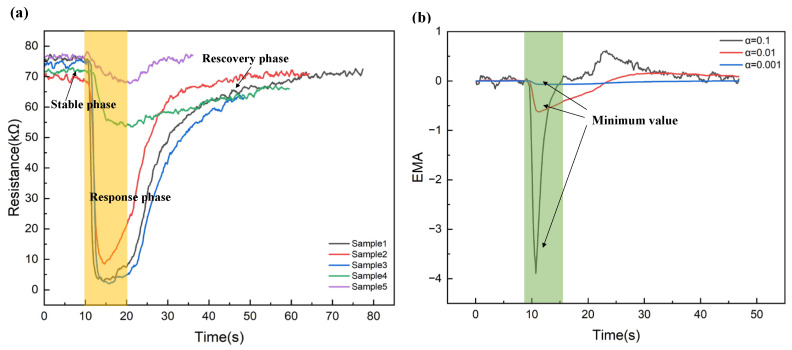
Schematic diagram of two different feature extraction methods; (**a**) A sensor measures the raw resistance of five samples and divides them into three response states; (**b**) EMA curves converted from raw data.

Each sensor provides two steady-state features and three transient features in the extracted data [28]. Specifically, the steady-state feature is defined as the difference between the maximum resistance change observed during the extraction response phase and the baseline resistance during the original response process, as shown in Figure 3a. The calculation is as follows:

(1) $$∆R=\underset{i}{max}r[i]-\underset{i}{min}r[i]$$

Another steady-state characteristic is its normalized value, calculated as follows:

(2) $$\left\|\Delta R\right\|=\frac{\underset{i}{max}r[i]-\underset{i}{min}r[i]}{\underset{i}{min}r[i]}$$

where i∈ [0, T] is the discrete time and r[i] is the time curve of the sensor resistance when gas is present in the test chamber. Three transient features were identified to represent the sensor’s response in addition to the two steady-state features. The discrete time series was converted into scalar values using the Exponential Moving Average (EMA) [29], as shown in Figure 3b. The calculation formula is as follows:

(3) $$ema_{\alpha }[i]=(1-\alpha )ema_{\alpha }[i-1]+\alpha (r[i]-r[i-1])$$

Among them, α∈ [0, 1] is a smoothing parameter and set $ema_{\alpha }[0]=0$. Set the smoothing parameters to 0.1, 0.01, and 0.001, respectively. Three EMA curves are extracted from the original data’s baseline and response stages, and the curves’ minimum values are selected to obtain the three characteristic values.

### 2.4. ReliefF Algorithm and Feature Alignment

#### 2.4.1. ReliefF Algorithm

The ReliefF algorithm is a machine learning algorithm commonly used for feature selection [30], with the primary goal of identifying and selecting the features with the highest correlation with the target variable from the original feature set. This algorithm was first proposed by Kira and Rendell in 1992 and has since undergone multiple improvements and extensions, becoming one of the essential tools in feature selection. The ReliefF algorithm retrieves k nearest neighbor samples (near Hits) of sample R from the sample set of the same type as R after randomly selecting one sample R from the training sample set each time when dealing with multi-class problems. The k nearest-neighbor samples (near-misses) are obtained from the sample set of various classes of each R. Every feature’s weight is updated [31]. The pseudo code is shown in Algorithm 1.
**Algorithm 1** Pseudocode for the ReliefF method for optimizing sensor arrays**Input:** for each training instance a vector of attribute values and the class value **Output:** the vector W of estimations of the qualities of attributes **Procedure:** 1: set all weights $W\left(A\right)$ = 0.0; 2: **for** i =1 **to** m **do begin**  randomly select an instance $R_{i}$  find k nearest hits $H_{j}$;  **for** each class C ≠ class($R_{i}$) **do**    from class C find k nearest misses $M_{j}\left(C\right)$; 3: **for** each featrue A =1 **to** a **do** $W(A)=W(A)-\Sigma_{j=1}^{k}\mathrm{diff}\;\left(A,R_{i},H_{j})/(m*k\right)+\;\;\Sigma_{C\notin \mathrm{class}\;\left(R_{i}\right)}\left[\frac{p\left(C\right)}{1-p\left(\mathrm{class}\;\left(R_{i}\right)\right)}\Sigma_{j=1}^{k}\mathrm{diff}\;\left(A,R_{i},M_{j}\left(C\right)\right)\right]/(m*k);$
4: **end;**

In the above equation, $diff\left(A,{\;l}_{1},{\;l}_{2}\right)$ represents the difference between samples $l_{1}$ and $l_{2}$ on feature A ($e.g{\;l}_{1},{\;l}_{2}$), $M_{j}\left(C\right)$ represents the jth nearest neighbor sample in class C∉class (R). As follows:

(4) $$diff(A,l_{1},l_{2})=\left\{\begin{array}{ll} \frac{\left|l_{1}[A]-l_{2}[A]\right|}{max(A)-min(A)} & \;\mathrm{If}\;A\;\mathrm{Is}\;\mathrm{Continuous}\;\\ 0 & \;\mathrm{If}\;A\;\mathrm{Is}\;\mathrm{Discrete}\;\mathrm{And}\;l_{1}[A]=l_{2}[A]\\ 1 & \;\mathrm{If}\;A\;\mathrm{Is}\;\mathrm{Discrete}\;\mathrm{And}\;l_{1}[A]\neq l_{2}[A] \end{array}\right.$$

In this article, we represent each sensor using normalized steady-state features extracted from sensor response curves and employ feature selection to screen materials.

#### 2.4.2. FA Feature Alignment Algorithm

The SA (subspace alignment) statistical feature alignment method mainly transforms and aligns the statistical features of the data. The aligned data can be learned using traditional machine learning methods to construct classifiers. The SA method is a representative achievement among them. The objective function of SA is as follows:

(5) $$F(P)={∥M_{s}P-M_{t}∥}_{F}^{2}$$

The value of transformation M is:

(6) $$P^{★}=arg\;\underset{P}{min}\;(F(P))$$

Among them, $M_{s}$, $M_{t}$ is the projection of feature data $X_{S}=\left[x_{S}^{1},\ldots ,x_{S}^{N_{S}}\right]\in R^{d\times N_{S}}$ in the source domain and feature data $X_{T}=\left[x_{T}^{1},\ldots ,x_{T}^{N_{T}}\right]\in R^{d\times N_{T}}$ in the target domain, respectively. SA aligns feature data by aligning subspace coordinate systems.

To align the features of two domains more intuitively and simply, we adopt the method of direct feature alignment, and we name this method FA (Feature Alignment). To obtain the change matrix P, some transfer samples from two domains are required. Randomly select two feature samples from each class in the source domain as the transfer samples $M_{S}=\left[m_{S}^{1},\ldots ,m_{S}^{N_{m}}\right]\in X_{S}$. Select feature samples with the same number but different distribution as transfer samples $M_{T}=\left[m_{T}^{1},\ldots ,m_{T}^{N_{m}}\right]\in X_{T}$ in the target domain. Based on SA, a regularization term $\lambda ∥P{∥}_{2}^{2}$ is added, a non-negative hyperparameter used to control the influence of the regularization term [32]. The calculation formula for the transformation matrix P is as follows:

(7) $$\underset{P}{min}\;{∥M_{S}-M_{T}P∥}_{2}^{2}+\lambda ∥P{∥}_{2}^{2}$$

The solving equation can be optimized as follows:

(8) $$P={\left({\left(M_{T}\right)}^{T}\cdot \left(M_{T}\right)+\lambda I\right)}^{-1}\cdot {\left(M_{T}\right)}^{T}\cdot \left(M_{S}\right)$$

By transformation matrix P, the target domain feature $X_{T}^{\prime }$=$X_{T}P$ after the change can be calculated. By using a Euclidean distance, the average offset of each class can be calculated using the following formula:

(9) $${ED}_{k}=\frac{\sqrt{\sum_{i=1}^{d_{k}}\;\sum_{j=1}^{N_{k}}{\left(x_{Tij}-X_{Tij}^{\prime }\right)}^{2}\;}}{d_{k}N_{k}}k=1,\ldots ,5$$

Among them, ${ED}_{k}$ represents the Euclidean distance difference of the kth dataset before and after FA. $d_{k}$ represents the number of features in the kth dataset. $N_{k}\;$represents the number of samples in the kth dataset. $x_{Tij}$ represents the value of the jth sample of the ith feature in the original dataset. $X_{Tij}^{\prime }$ represents the value of the jth sample of the ith feature after performing FA on the original dataset. Here are the calculation steps for this formula:

For each dataset k, calculate the value $x_{Tij}$ for each sample j on each feature i and the value $X_{Tij}^{\prime }$ after FA.

Sum the squares of the differences for all features i and all samples j to obtain the total sum of squared errors.

Compute the square root of the total sum of squared errors for the kth datasets, then divide by the product of the number of samples and the number of features to calculate the Euclidean Distance (${ED}_{k}$) error.

This formula is used to measure the degree of difference between the dataset after FA and the original dataset. A smaller ${ED}_{k}$ value indicates that the two datasets are closer, implying better calibration.

**Figure 4:**
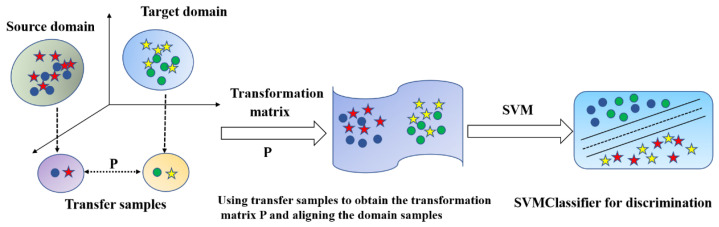
The schematic diagram of FA feature alignment.

The schematic diagram of FA feature alignment is shown in Figure 4, and its pseudo-code is shown in Algorithm 2.
**Algorithm 2** The FA method for feature alignment**Input:** The source domain feature matrix $X_{S}=\left[x_{S}^{1},\ldots ,x_{S}^{N_{S}}\right]\in R^{d\times N_{S}}$ The source domain label matrix $T_{S}=\left[T_{S}^{1},\ldots ,T_{S}^{N_{S}}\right]\in R^{d\times N_{S}}$ The target domain feature matrix $X_{T}=\left[x_{T}^{1},\ldots ,x_{T}^{N_{T}}\right]\in R^{d\times N_{T}}$ The transfer samples of source domain $M_{S}=\left[m_{S}^{1},\ldots ,m_{S}^{N_{m}}\right]\in X_{S}$ The transfer samples of target domain $M_{T}=\left[m_{T}^{1},\ldots ,m_{T}^{N_{m}}\right]\in X_{T}$ The regularization parameter λ **Output:** Transformation matrix P New target domain $X_{T}^{\prime }$ Recognition accuracy (%) of unlabeled data in new target domain **Procedure:** 1: Calculate the basis transformation matrix P according to (8) 2: Calculate the **new target domain**
$X_{T}^{\prime }$ according to $X_{T}^{\prime }\;$=$X_{T}P$ 3: Calculate the predicted output of unlabeled samples of new target domain $\;T_{T}\;$← SVMClassifier ${(X}_{S},T_{S},X_{T}^{\prime }$) 4: Calculate the recognition accuracy (%) of unlabeled data in new target domain

## 3. Results and Discussion

### 3.1. Microstructure Characteristics of Sensing Membranes

**Figure 5:**
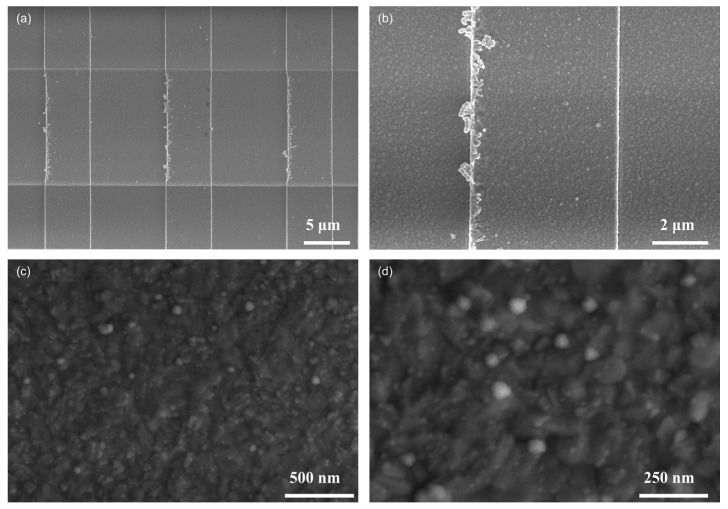
SEM images of SnO_2_ at different multiples. (**a**,**b**) Partial schematic diagram of SnO_2_ sensor; (**c**,**d**) Detailed image of SnO_2_ thin film.

Figure 5 presents the SEM images of pure SnO_2_ at various magnifications. The surface morphology of the pure SnO_2_ film exhibits a granular texture, with the film layer appearing relatively dense and uniform. Higher-magnification images reveal the presence of numerous micro-wrinkle structures on the material’s surface. Consequently, the increased contact area between the material and the gas enhances its gas sensitivity performance. The atomic distribution and composition of oxygen (O) and tin (Sn) in pure SnO_2_ thin films were analyzed using Energy Dispersive X-ray Spectroscopy (EDS), as illustrated in Figure S5. The analysis revealed that the atomic percentage of oxygen was 65.4%, while that of tin was 18.76%. Furthermore, the atomic percentage of silicon (Si) was determined to be 15.84%.

**Figure 6:**
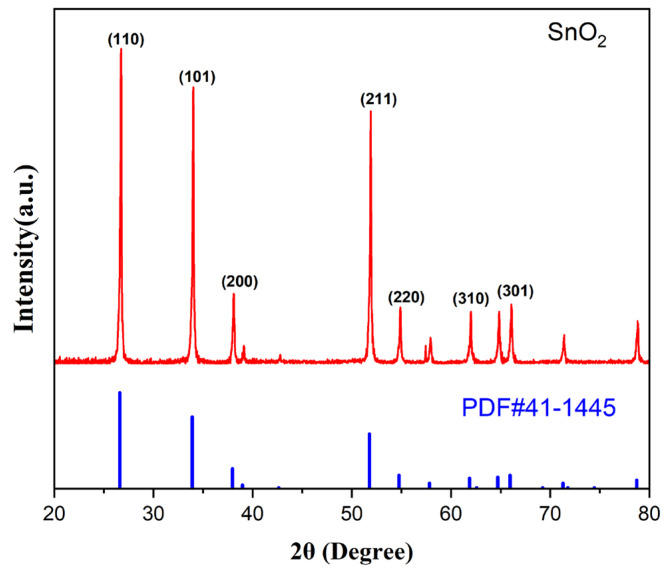
XRD patterns of pure SnO_2_.

Figure 6 presents the XRD characterization of pure SnO_2_. The distinct diffraction peaks observed at 26.73°, 34.01°, and 51.90° correspond to the (110), (101), and (211) planes of SnO_2_, respectively, as referenced by PDF card No. 41-1445. These findings confirm that the sample exhibits a rutile–tetragonal cassiterite structure.

### 3.2. Using the ReliefF Algorithm to Select Sensor Materials

**Figure 7:**
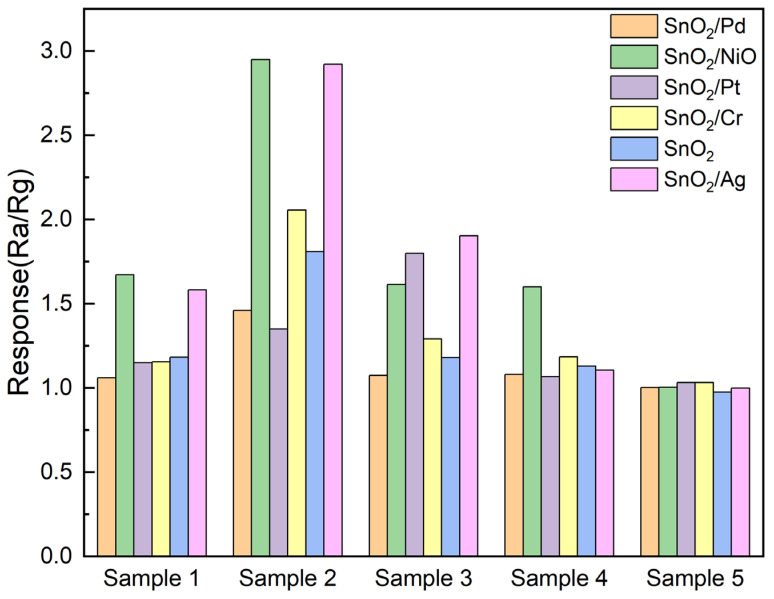
Sensor response to five pharmaceutical products.

The response values of six SnO_2_-based sensors to four pharmaceutical compounds are presented in Figure 7. The SnO_2_/NiO sensor and the SnO_2_/Ag sensor exhibited the highest response to the drugs in Sample 2, with response values of 2.9 and 2.8, respectively. The distinct responses exhibited by each sensor towards the drugs facilitate the classification experiments. The real-time response value of the six sensors to four drug samples is shown in Figure S6. Among them, due to the weak response of the sensors to Huanglian Shangqing Tablets, no response graph was made. Nevertheless, this weak response is still regarded as a distinguishing characteristic of this particular drug.

**Figure 8:**
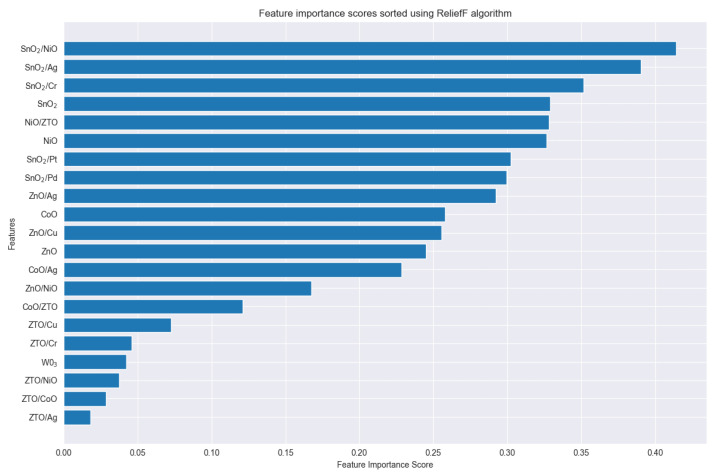
Ranking of importance scores for 21 materials using the ReliefF algorithm.

The sensor pool consists of the 6 SnO_2_-based sensors mentioned above and 15 different sensors provided by our research team. The specific types can be found in Table S1. Within this pool of 21 sensors with diverse materials, we apply the ReliefF algorithm to obtain classification contribution scores for each material, sorting them in descending order as shown in Figure 8. Based on this ranking, it is apparent that sensors incorporating SnO_2_ exhibit a superior performance in classification tasks, whereas sensors containing ZTO demonstrate comparatively lower classification efficacy.

**Figure 9:**
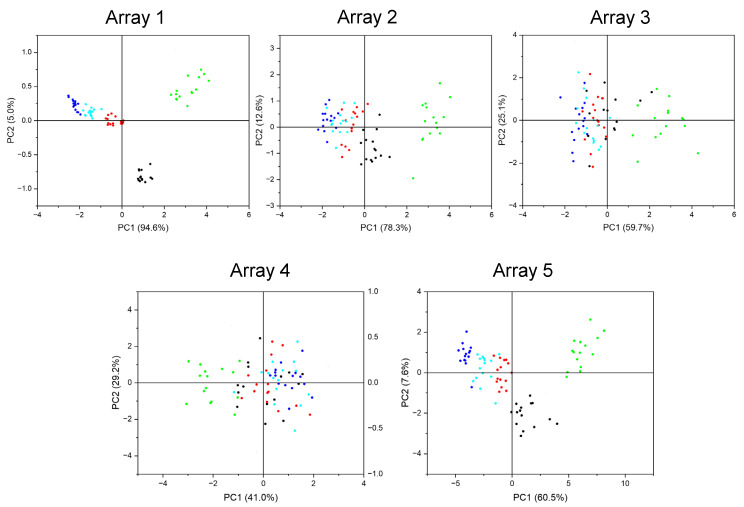
Comparison of different sensor arrays through PCA graphs.

To evaluate the efficacy of the proposed ranking system, we will designate the array comprising the top four sensors (ranked first to fourth) as Array 1, and the array containing sensors ranked fifth to eighth as Array 2. Similarly, the array consisting of sensors ranked fourteenth to seventeenth will be referred to as Array 3, while the array including sensors ranked eighteenth to twenty-first will be designated as Array 4. Lastly, the array encompassing all sensors will be termed Array 5. We will employ Principal Component Analysis (PCA) to visualize and assess their classification performance. The results are presented in Figure 9. Analyzing the outcomes of the PCA method reveals that arrays composed of the highest-ranked sensors demonstrate a superior classification performance. Conversely, arrays consisting of the lowest-ranked sensors exhibit an inferior classification performance. This comparison between Array 1 and Array 5 highlights a decline in the classification performance when all sensors are aggregated into a single array (Array 5). This observation substantiates that an increased number of sensors does not inherently enhance the classification performance. Indeed, it may result in diminished classification accuracy due to redundant information.

### 3.3. Using FA Algorithm for Model Calibration

To verify the FA algorithm, which can enhance model sharing among distinct electronic nose devices, we constructed three electronic nose devices with identical material compositions. We tested five drugs using three sensor arrays, with each experiment repeated 15 times. Consequently, each sensor array generated 75 raw data points. These data points were preprocessed to yield a dataset of 75 × 20 (each sensor extracted five features, and each array comprised feature data from four sensors). The feature data from the three sensor arrays were designated as Data 1, Data 2, and Data 3.

**Figure 10:**
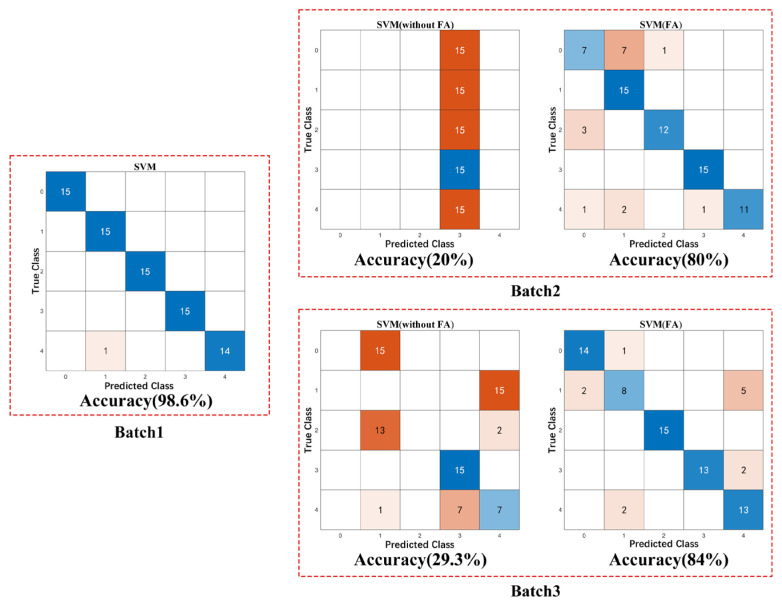
Comparing the classification performance with and without FA using SVM confusion matrix graph.

Initially, we conduct K-fold Cross Validation on Dataset 1 to obtain training results using the Support Vector Machine (SVM) algorithm, which will be presented as Batch 1 via a confusion matrix. The accuracy result is 98.6% (95% confidence level, with a confidence interval of 92.78–99.97%). Subsequently, we employ Dataset 1 as the source domain for model training, while Datasets 2 and 3 serve as the target domains for model testing. The test results should be compared between the scenarios with Feature Augmentation (FA) and without FA, and these results will be depicted as Batch 2 and Batch 3, respectively. The confusion matrix is illustrated in Figure 10. The analysis of the confusion matrix for Batch 1 indicates that the sensor array demonstrates a robust classification performance for drug detection. However, the confusion matrix results for Batches 2 and 3 reveal that when Data 1 is employed as the training set, and Data 2 and Data 3 are utilized as the testing sets, the classification accuracy declines to 20% and 29.3%, respectively. This discrepancy can be attributed to variations in the electronic noses, which hinder the effective transferability of the algorithmic model.

Following the alignment of Data 2 and Data 3 with Data 1 via the FA algorithm, the accuracy increased to 80% (95% confidence level, with a confidence interval of 69.17–88.35%) and 84% (95% confidence level, with a confidence interval of 73.72–91.45%), respectively, when Data 2 and Data 3 were used to validate the model trained on Data 1. Consequently, through post-calibration through the FA algorithm, these three distinct electronic nose systems can utilize a unified model rather than requiring individual models for each system. To verify the effectiveness of the FA algorithm, we compared three calibration algorithms (UDS, DS-L1R, and DS-PLS2) using the same method as described above, and compared the accuracy before and after calibration using Batch 2 and Batch 3. The results are shown in Table 2.

From the results, UDS is not suitable for multivariate calibration models. After calibration with DS-L1R and DS-PLS2, the accuracy has been improved to some extent, but the effect is still not as good as FA, thus confirming the effectiveness of the FA feature alignment algorithm. To understand the changes before and after calibration for each sample, we use the Euclidean distance for calculation.

As demonstrated in Table 3, the average offset of each class feature post-alignment was computed using the Euclidean distance metric. The data presented in Table 3 indicate a significant disparity in feature data between the target domain and the source domain prior to the application of the FA algorithm. In the post-application of FA, the feature data for most categories in both domains exhibit increased similarity, although a minor subset of categories shows a slight divergence. These results substantiate the efficacy of FA in aligning feature data within the context of electronic nose datasets.

## 4. Conclusions

In this work, we utilize the drug classification experiment as a case study. Detailed descriptions are provided regarding the preparation of MEMS gas sensors, the design of sensor packaging and peripheral circuits, and the construction process of the electronic nose apparatus. Within the circuit design, PWM waves are employed to modulate various heating voltages as required through the pulse heating technique. The stability of the heating voltage is further enhanced by the feedback circuit design, thereby augmenting the overall stability of the sensor. The ReliefF algorithm is used to select the most effective sensor types from the available sensor pool based on their respective contribution values, thereby optimizing the sensor array. Subsequently, the FA algorithm is applied to enable three electronic nose devices with poor consistency to share a common model. By training a model on data derived from one sensor array and subsequently testing it on data from the other two sensor arrays, the classification accuracy was enhanced from 20% to 80% and from 29.3% to 84%, respectively. These results underscore the efficacy of the FA in addressing the transfer calibration problem among electronic nose devices.

## Figures and Tables

**Table 1:** Summary of different drugs.

Category Number	Drugs Name
Sample 1	Bismuth Potassium Citrate Tablets
Sample 2	Compound Eosinophil–Lactobacillus Tablets
Sample 3	Banlangen granules
Sample 4	Lianhua Qingwen Capsule
Sample 5	Huanglian Shangqing Tablets

**Table 2:** Results of different calibration algorithms.

Algorithms	Batch 2 (Accuracy)	Batch 3 (Accuracy)
UDS	20% → 26.7%	29.3% → 28%
DS-L1R	20.0% → 54.7%	29.3% → 61.3%
DS-PLS2	20.0% → 73.3%	29.3% → 77.3%
FA	20.0% → 80.0% (95% CI: 69.17–88.35%)	29.3% → 84.0% (95% CI: 73.72–91.45%)

**Table 3:** Calculate the average offset of each class feature after using FA feature alignment using Euclidean distance.

Sample	Batch2	Batch3
Sample 1	8.907 → 2.577	11.855 → 2.029
Sample 2	4.956 → 1.083	3.300 → 1.462
Sample 3	15.703 **→** 2.174	11.269 **→** 2.376
Sample 4	0.856 → 1.183	1.514 → 1.183
Sample 5	2.795 → 1.348	1.822 → 2.048

## Data Availability

Data are contained within the article.

## Supplementary Materials

The following supporting information can be downloaded at: https://www.mdpi.com/article/10.3390/s25051480/s1, Figure S1: The readout circuit and pulse heating method of the MEMS gas sensor; (a) The packaged chip and the peripheral readout circuitry; (b) A feedback circuit for PWM wave control of heating temperature; (c) Voltage conversion circuits; Figure S2: Physical diagram of the electronic nose system; Figure S3: Gas sensor array monitoring and analysis system based on LabVIEW. This host computer can detect four pieces of channel data in real time and save them into an EXCEL format; Figure S4: Physical diagram of five classes of drugs; (a) Bismuth Potassium Citrate Tablets; (b) Huanglian Shangqing Tablets; (c) Banlangen granules; (d) Lianhua Qingwen Capsule; (e) Compound Eosinophil–Lactobacillus Tablets.; Figure S5: EDS images of pure SnO_2_; Figure S6: Sensor response to four pharmaceutical products; (a) Bismuth Potassium Citrate Tablets; (b) Huanglian Shangqing Tablets; (c) Banlangen granules; (d) Compound Eosinophil–Lactobacillus; Table S1: Summary of 21 different material sensors; Table S2: Parameters of SVM; Table S3: Parameters for material preparation.
