# Supplementary material for: Enhancing E-Nose Performance via Metal-Oxide Based MEMS Sensor Arrays Optimization and Feature Alignment for Drug Classification
Source: Sensors (Basel). 2025 Feb 28;25(5):1480. doi: 10.3390/s25051480 (PMC11902490; doi:10.3390/s25051480)
Supplement: Supplementary file 1 [file sensors-25-01480-s001.zip › sensors-3454020-supplementary.pdf]

Enhancing E-Nose Performance via Metal-Oxide Based MEMS Sensor Arrays Optimization and Feature Alignment for Drug Classification

Ruiwen Kong [1,2], Wenfeng Shen [2,3,4,5,*], Yang Gao [5,6], Dawu Lv [2,3], Ling Ai [2,3], Weijie Song [2,3,*] and Ruiqin Tan [1,*]

[1] Faculty of Electrical Engineering and Computer Science, Ningbo University, Ningbo 315211, China; kongruiwen@nimte.ac.cn
[2] Center of Materials Science and Optoelectronics Engineering, University of Chinese Academy of Sciences, Beijing 100049, China; lvdawu@nimte.ac.cn (D.L.); ailing@nimte.ac.cn (L.A.)
[3] Zhejiang Provincial Engineering Research Center of Energy Optoelectronic Materials and Devices, Ningbo Institute of Material Technology and Engineering, Chinese Academy of Sciences, Ningbo 315201, China
[4] CS-Microsensor (Ningbo) Technology Co. Ltd., Ningbo 311121, China
[5] Research and Manufacturing Base of Olfactory Sensors in China Sensing Valley, Bengbu 233040, China; yanggao@csmsn.com
[6] Hangzhou Dianzi University, Shanghai 201713, China
* Correspondence: wfshen@nimte.ac.cn (W.S.); tanruiqin@nbu.edu.cn (R.T.);

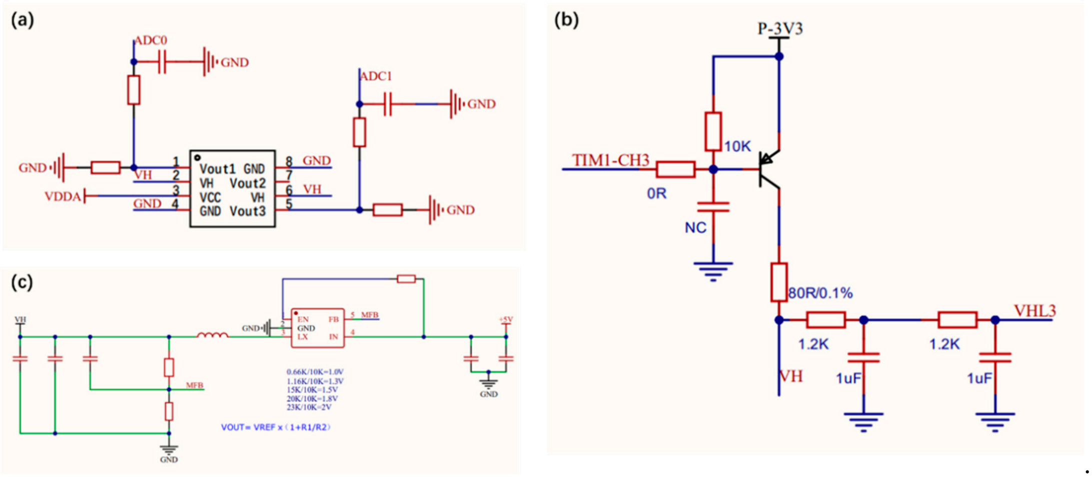

Figure S1. The readout circuit and pulse heating method of the MEMS gas sensor; (a) The packaged chip and the peripheral readout circuitry; (b) A feedback circuit for PWM wave control of heating temperature; (c) Voltage conversion circuits.

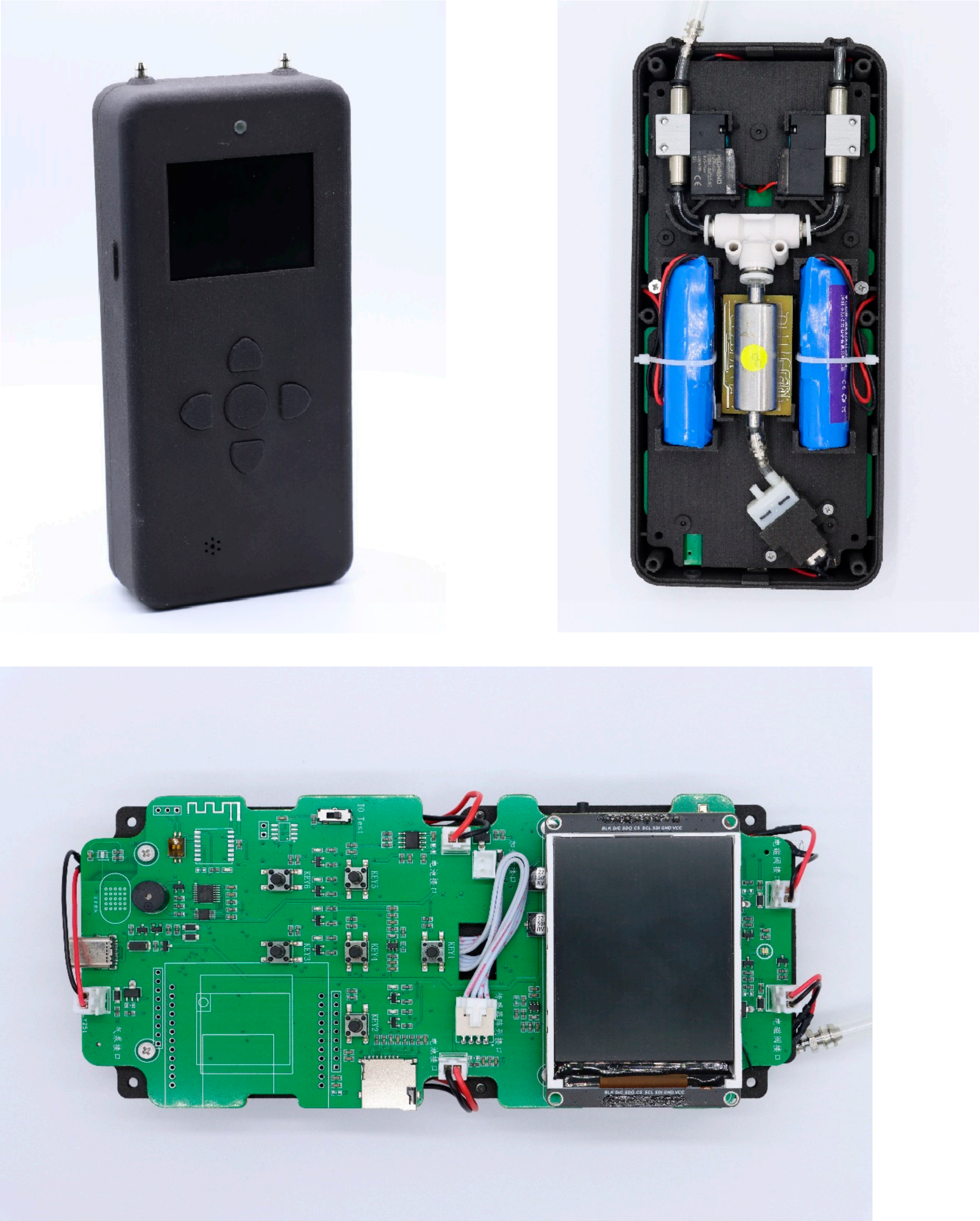

Figure. S2. Physical diagram of the electronic nose system.

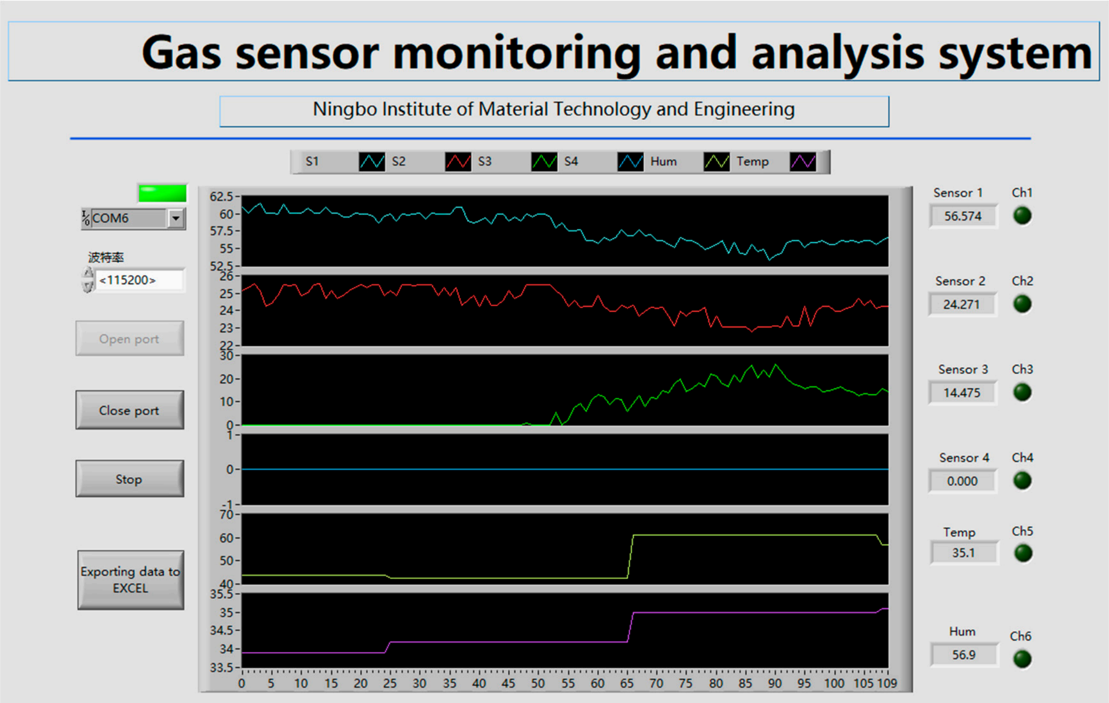

Figure. S3. Gas sensor array monitoring and analysis system based on LabVIEW. This host computer can detect four channel data in real time and save it into an EXCEL format table.

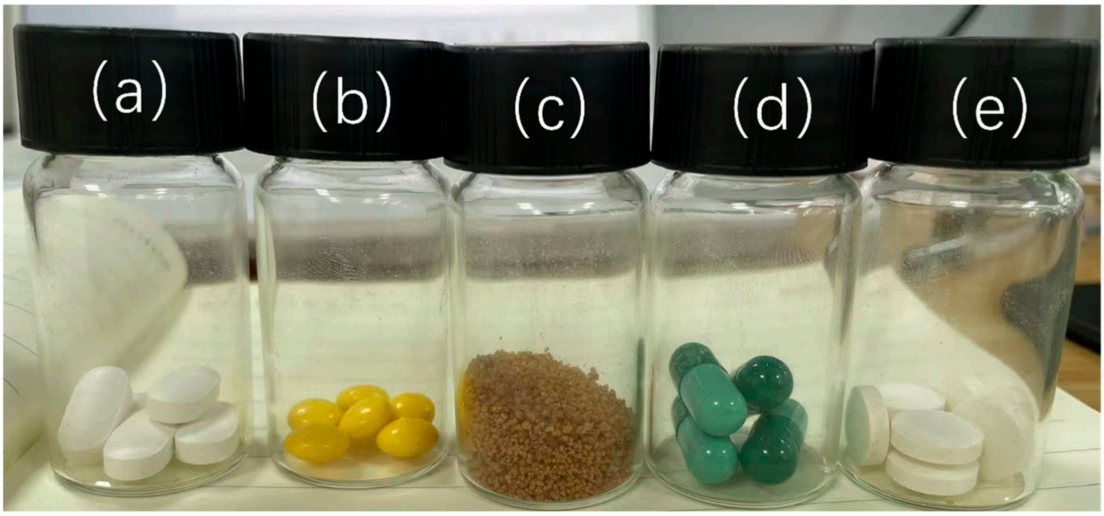

Figure. S4. Physical diagram of five classes of drugs; (a) Bismuth Potassium Citrate Tablets; (b) Huanglian Shangqing Tablets; (c) Banlangen granules; (d) Lianhua Qingwen Capsule; (e) Compound Eosinophil-Lactobacillus Tablets.

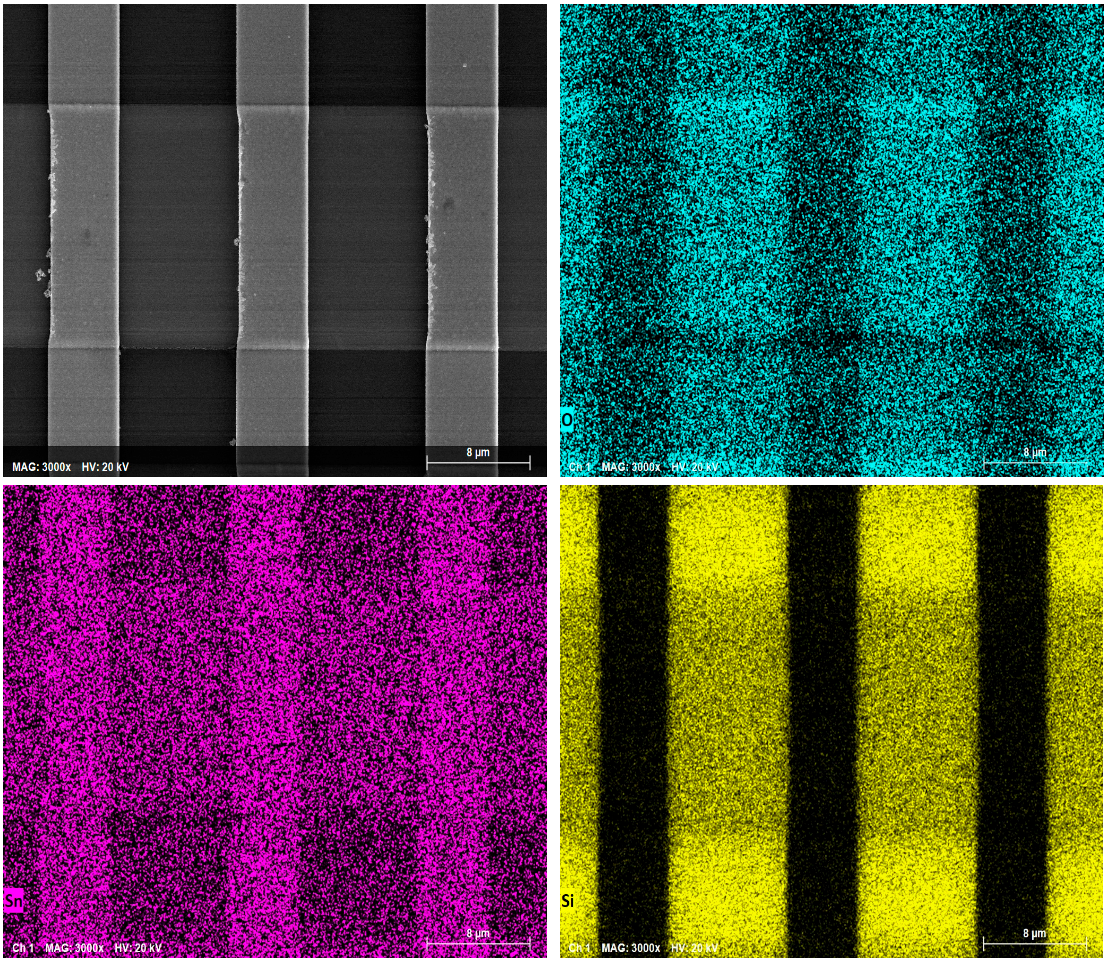

Figure. S5 EDS images of pure SnO_2.

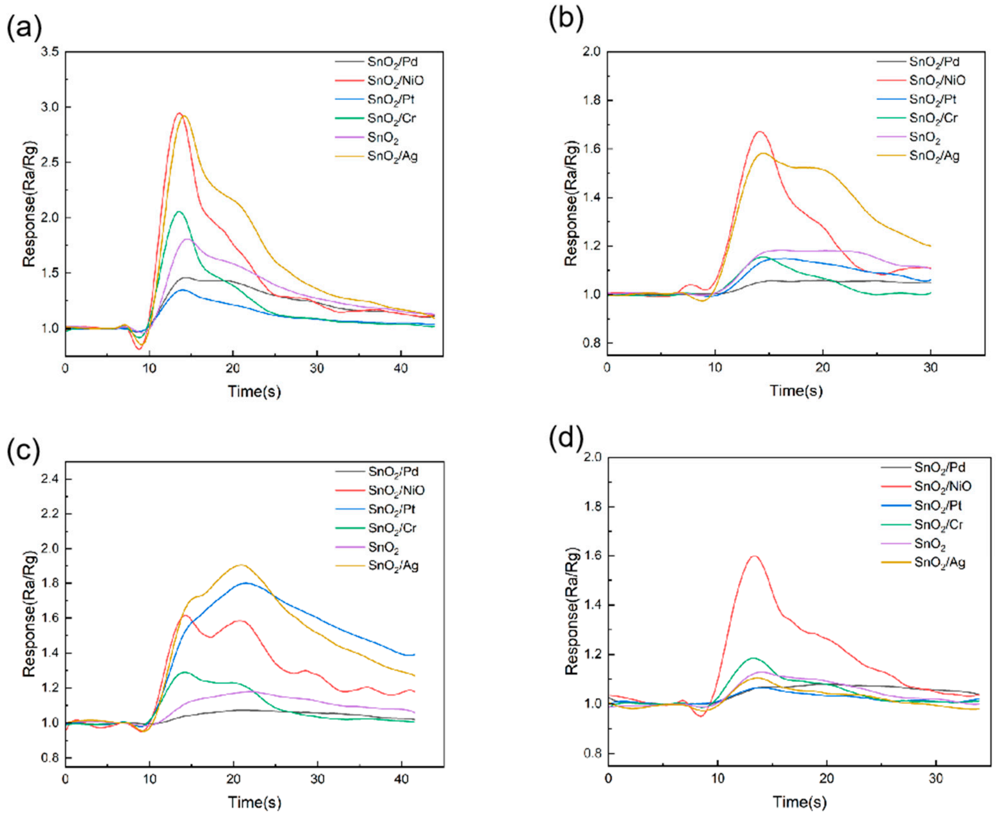

Figure. S6. Sensor response to four pharmaceutical products; (a) Bismuth Potassium Citrate Tablets; (b) Huanglian Shangqing Tablets; (c) Banlangen granules; (d) Compound Eosinophil-Lactobacil.

Table S1. Summary of 21 different material sensors.

Serial number	Gas sensitive materials	Doping content
1	SnO_2	
2	SnO_2/Ag	0.7%
3	SnO_2/Cr	0.6%
4	SnO_2/Pd	0.5%
5	SnO_2/Pt	0.6%
6	SnO_2/NiO	0.5%
7	ZnO	
8	ZnO/NiO	0.6%
9	ZnO/Ag	0.7%
10	ZnO/Cu	0.5%
11	ZTO/Cr	0.7%
12	ZTO/Ag	0.7%
13	ZTO/CoO	0.6%
14	ZTO/NiO	0.5%
15	ZTO/Cu	0.7%
16	CoO	
17	CoO/ZTO	0.6%
18	CoO/Ag	0.6%
19	NiO	
20	NiO/ZTO	0.7%
21	WO_3	

Table S2 Parameters of SVM

Kernel function	Gaussian, Linear, Quadratic, Cubic
Box constraint level	log-scaled in the range [0.001,1000]
Kernel scale	log-scaled in the range [0.001,1000]
Standardize data	Ture

Table S3 Parameters for material preparation

Material	Thin Films	RF Power(w)	Flow rate (sccm)	Deposition Time(minute)
SnO_2	SnO_2	150	37.4	120
SnO_2/Ag	SnO_2	150	35.1	120
	Ag	5	34.3	3
SnO_2/Cr	SnO_2	150	35.1	120
	Cr	3	38.2	3
SnO_2/NiO	SnO_2	150	35.1	120
	NiO	150	37.4	20
SnO_2/Pt	SnO_2	150	35.1	120
	Pt	5	34.3	3
SnO_2/Pd	SnO_2	150	35.1	120
	Pd	5	34.3	3
